# Hepatitis B Triple Panel Testing Implementation in the Obstetric Care Setting: Unique Predictors of Hepatitis B Virus Vaccine Immunity, Exposure, and Positivity

**DOI:** 10.1093/ofid/ofae632

**Published:** 2024-10-23

**Authors:** Natalia Schmidt, Jeanette Rios, Lauren Alpert, Anna Mageras, Whitney Lieb, Tatyana Kushner

**Affiliations:** Department of Internal Medicine, Icahn School of Medicine at Mount Sinai, New York, New York, USA; Icahn School of Medicine at Mount Sinai, New York, New York, USA; Icahn School of Medicine at Mount Sinai, New York, New York, USA; Division of Liver Diseases, Icahn School of Medicine at Mount Sinai, New York, New York, USA; Department of Obstetrics, Gynecology and Reproductive Science, Icahn School of Medicine at Mount Sinai, New York, New York, USA; Division of Liver Diseases, Icahn School of Medicine at Mount Sinai, New York, New York, USA

**Keywords:** HBV, HBV immunization, HBV triple panel, hepatitis B, pregnancy, prenatal

## OBJECTIVE

In March 2023, the Centers for Disease Control and Prevention (CDC) released updated hepatitis B virus (HBV) screening guidelines, recommending universal HBV screening with a triple panel test (HBsAg, anti-HBs, and anti-HBc) for all adults, at least once, after age 18 years, including those who are pregnant [1]. The American College of Obstetricians and Gynecologists (ACOG) subsequently released its updated HBV screening recommendations in August 2023 [2], in accordance with the CDC [1], recommending universal triple panel testing in pregnancy, in contrast to prior recommendations for HBsAg testing only. Current guidelines also recommend HBV vaccination during pregnancy in those who are not immune [2–6]. HBV triple panel screening was quickly implemented in our large urban academic center, where an estimated 15 000 pregnancy deliveries are performed annually. Following guideline changes, 2 large hospital-based obstetrics practices, which perform between 1000 and 1400 pregnancy deliveries per year each, included all 3 tests into a prenatal laboratory order set with the addition of the anti-HBc test in February 2024. Our study aims to evaluate HBV screening rates and predictors in pregnancy after implementing HBV triple panel screening, with particular interest in anti-HBs and anti-HBc results, not previously routinely tested in pregnancy.

### Patient Consent Statement

Institutional review board approval was obtained for this study. This study did not include factors necessitating patient consent.

### Study Design

After obtaining board approval, we conducted a retrospective chart review of women aged >18 years with at least 1 documented pregnancy encounter and HBV test result across all obstetric clinics at our academic center between August 2021 and March 2024 (ie, 2 years before the guideline change and 2 months after triple panel implementation at 2 obstetric clinics). Testing for HBsAg, anti-HBc, and anti-HBs before and after implementation was evaluated over time, and chi-square tests were used to evaluate factors associated with positive test results.

## RESULTS

**Figure 1.**
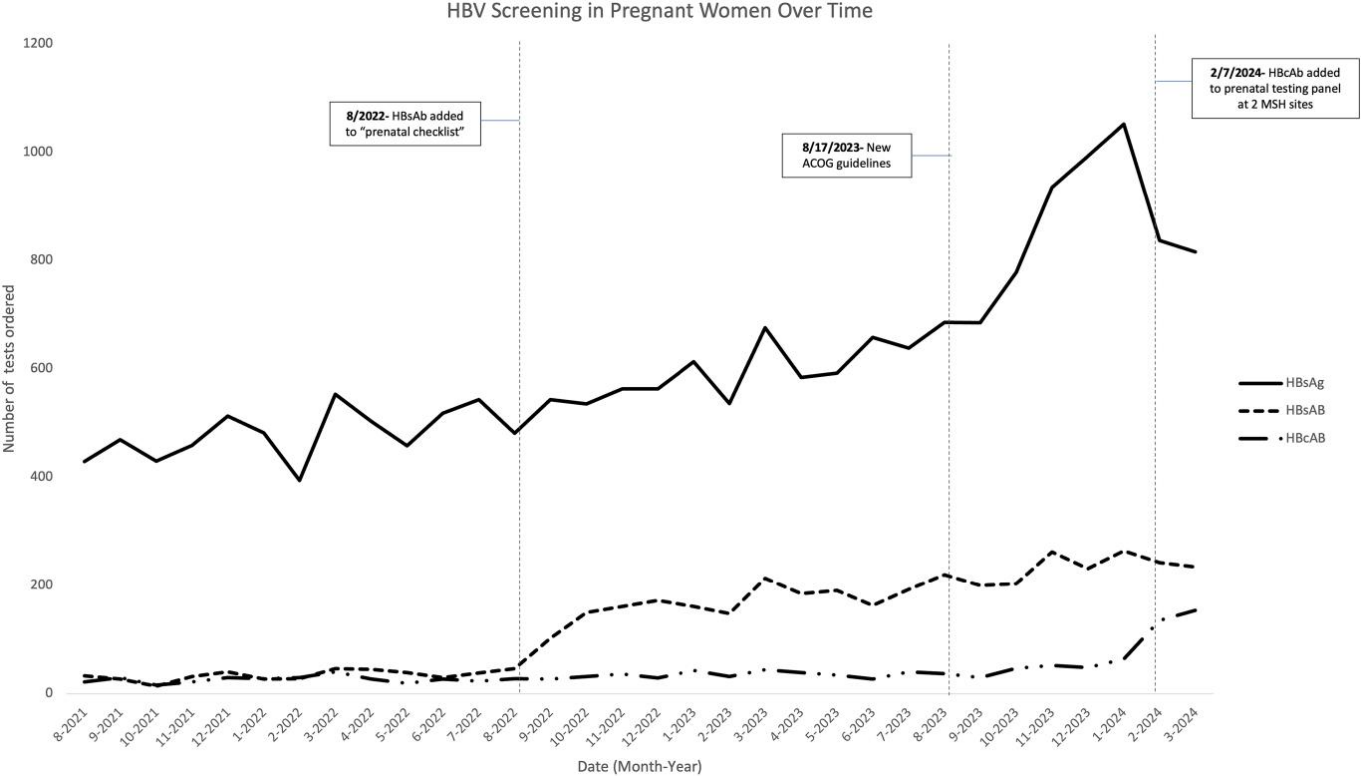
Most recent HBsAg, HBsAb, and HBcAb tests performed between August 2021 and March 2024 for women who had at least 1 pregnancy encounter during this period across all Mount Sinai prenatal clinics, plotted over time. Dashed lines indicate 3 time points: addition of HBsAb to a “prenatal checklist” to 2 MSH clinics, ACOG releases new triple screen guidelines, and addition of all 3 HBV tests to a prenatal laboratory order set at 2 MSH clinic sites. ACOG, American College of Obstetricians and Gynecologists; HBV, hepatitis B virus; MSH, Mount Sinai Hospital.

A total of 19 499 individuals met our criteria, with a median age of 34 years (IQR, 30–38); 40% were White, 24% Hispanic, 15% Black, and 12% Asian. As shown in Figure 1, HBV testing among pregnant women increased with the implementation of triple screening, with the anti-HBs test added to 2 clinics' prenatal laboratory order set in August 2022, followed by the addition of anti-HBc to these practices' prenatal panel in February 2024. Across all clinic sites, 1566 patients (8%) had a complete triple panel test, with the highest rates among those who were Black (13%), followed by Hispanic (10%) and Asian (6%, *P* < .01). Higher rates of triple panel tests were seen among Medicaid patients as compared with other insurance types (11% vs 7%, *P* < .01). Individuals were also divided by the date of their HBsAg test, used as a surrogate for prenatal testing date, to better represent rates of triple screening pre- and postimplementation. Of the 1638 patients with an HBsAg test ordered after implementation in February 2024, 332 (20.3%) were triple screened, as opposed to 1234 (6.9%) of the 17 861 tested prior to implementation (*P* < .01).

Regarding test results across all clinics, 5038 patients (26%) were tested for anti-HBs, 28% of whom were nonreactive. Of the 1430 with nonreactive anti-HBs results, 394 (25%) were negative for all 3 HBV tests, suggestive of possible nonimmunity, with higher rates among Hispanic (32%) and Black (24%) patients as compared with those who were Asian (16%) and White (19%, *P* < .01). A total of 19 495 (99%) were tested for HBsAg: 80 (0.4%) had a reactive HBsAg, with highest rates among Asian patients as compared with all other races (1.4% vs 0.3%, *P* < .01). Of the 1779 (9%) who were tested for anti-HBc, 95 (5%) had a reactive anti-HBc result, with higher rates among Black (12%) and Asian (10%) women as compared with those who were White (1.5%) and Hispanic (1.4%, *P* < .01).

## CONCLUSION

Incorporating the HBV triple panel into prenatal visits offers an important opportunity to improve overall rates of HBV testing as well as immunization. Only 8% of our entire pregnancy cohort received triple testing before and after implementation, which includes patients from multiple satellite practices. We utilized the dates of the HBsAg test, as routinely used in pregnancy prior to the recent guideline changes, to represent the dates of prenatal appointments. In doing so, we identified a statistically significant increase in HBV triple screening in our cohort from 6.9% of those tested for HBsAg prior to implementation to 20.3% after the triple panel was added to 2 practices' prenatal testing panel. Despite this apparent increase in triple panel screening rates, nearly 80% of patients were still not being triple screened despite guideline changes and the addition of the HBV triple panel to 2 of our clinics' prenatal laboratory panel, indicating that triple panel screening guidelines are not being rapidly implemented across practices outside the hospital setting. These findings are in contrast to a relatively more rapid implementation of prenatal hepatitis C virus (HCV) testing following the CDC and US Preventive Services Task Force's updated guidelines in 2020, as demonstrated by Kaufman et al, who used Quest data to examine national rates of prenatal anti-HCV testing over time. Although not directly comparable to this study, given the shorter time frame of this study and differences in analysis, the anti-HCV test was included in up to 40% of prenatal testing approximately 1 year after guidelines were updated [7]. The lower rates of triple screening in our study can be explained, in part, by the inclusion of our entire pregnancy cohort, including numerous satellite practices and not just the 2 sites that were able to incorporate the triple testing into their prenatal testing panel. For reference, out of the approximately 15 000 pregnancy deliveries performed system-wide per year, these 2 clinics combined, though large, perform only 2200 to 2500 of these deliveries. However, this still highlights a need for improved implementation across all clinic practice sites. A consideration can be made for implementing the triple panel testing at the time of delivery so that patients who present for delivery from different practice sites all receive testing at the time of delivery.

Our results demonstrate higher rates of triple panel testing among Medicaid patients as compared with those with other insurance types. This finding reflects the patient population of the 2 hospital-based clinics that incorporated the triple panel into a prenatal order set, which serve predominantly Medicaid patients. However, this highlights differences and potential barriers to successful and efficient implementation of guideline-based changes such as these in satellite clinics, which tend to have nonuniform and more individualized approaches to prenatal care testing as compared with hospital-based clinics.

The addition of anti-HBs and anti-HBc tests can identify those who are nonimmune and those with prior exposure to HBV. Patients without documented completion of the HBV vaccine series, in addition to undetectable anti-HBs and anti-HBc, are considered nonimmune to HBV. Identifying nonimmune persons allows for counseling and efficient HBV vaccination, which is safe and effective in pregnancy [6]. According to the Advisory Committee on Immunization Practices (ACIP), as well as the CDC, the American Association for the Study of Liver Diseases, and ACOG, all pregnant women who are nonimmune to HBV, regardless of risk factors, should be vaccinated with Engerix-B, Recombivax HB, or Twinrix during pregnancy [2–6]. Although testing negative for anti-HBs and anti-HBc is suggestive of nonimmunity, it is important to note that anti-HBs titers wane over time after completion of HBV vaccine series, without the loss of protection against HBV; thus, some patients who are “triple negative” may actually still be immune [8]. Therefore, according to the ACIP, it is not recommended for patients with a documented complete HBV vaccine series to receive additional immunization or boosters despite having a negative anti-HBs result, except for certain high-risk populations who are at an increased risk (eg, health care workers, patients undergoing hemodialysis, patients who are immunocompromised) [9]. Recommendations regarding the management of “triple negative” patients in pregnancy are not explicitly addressed in the ACOG vaccine guidelines, though they are written in alignment with the ACIP recommendations [2]. This may however, require clarification with the implementation of the triple panel screening, given the prevalence of this scenario. Of note, almost 30% of our cohort tested negative for anti-HBs and anti-HBc, with higher rates in those who were Hispanic or Black, highlighting potential target communities for increasing HBV vaccine efforts. Yet, it is important to note that in this study, vaccination status was not included in data collection, and we are therefore unable to distinguish between those who are truly nonimmune and those with diminished anti-HBs titers. In the future, it would be of interest to determine the vaccination status of this cohort to define nonimmunity and identify the proportion of nonimmune individuals who were offered and amenable to the HBV vaccine during pregnancy, in response to this screening.

The most common consult received after implementation of triple panel testing pertained to the interpretation and actionable management in individuals who tested positive for anti-HBc, which was about 5% of our cohort. A positive anti-HBc result is indicative of a prior exposure to HBV that typically has either resolved (positive anti-HBs and anti-HBc, negative HBsAg) or resulted in chronic HBV infection (positive HBsAg and anti-HBc). A positive anti-HBc result can also be seen in isolation (with negative HBsAg and anti-HBs), indicating a false-positive test, a resolved remote infection in which anti-HBs levels have waned, or an occult HBV infection. Regardless of interpretation of the result, a true positive anti-HBc result notably carries a risk of HBV reactivation in the setting of immunosuppression [10, 11]. One important area for clarification in the future will be the management of those with an isolated anti-HBc in pregnancy and the possible role for repeat testing once postpartum to confirm no active infection.

At our center, we identified anti-HBc positive consults as an educational opportunity for the management and counseling of patients who test positive for anti-HBc. In our experience, patients are often surprised to find out that they have a positive anti-HBc result on testing, indicating an often unknown prior exposure. Pregnancy is frequently the first time that individuals learn about viral hepatitis, with 1 study reporting a new diagnosis of HBV in 33% of pregnancies included in the study [12]. Although it can cause unnecessary anxiety, it is important to report and explain this result to patients, even while pregnant, as it will come up in the future if primary care physicians are following triple panel testing guidance. There were variable responses from patients who were anti-HBc positive, including some that were anxious about this new knowledge. Consultation with a liver specialist and/or discussion with the obstetrics and gynecology provider was helpful in providing the necessary education regarding anti-HBc–positive status. To aid in patient counseling, we developed a smartphrase (Appendix 1), which explains that a positive result does not indicate an active HBV infection, a risk of mother-to-child transmission, or a need for vaccination. It also highlights the importance of disclosing this result prior to starting any immunosuppressive medication, given the possible risk of HBV reactivation.

In March 2024, the World Health Organization released updated HBV guidelines, which include recommendations for universal reflex hepatitis D virus (HDV) testing, when available, for those who test positive for HBsAg, which was 0.4% of our cohort [13]. Following these guidelines, our institution began the process of implementing system-wide HDV reflex testing, including the obstetric clinics, where this is being incorporated into the prenatal testing panel. Though not included in this study, it would be of interest to investigate rates of HDV reflex testing and positivity in the prenatal care setting.

Pregnancy is a unique period, in which many women have regular access to health care for the first time, therefore offering an important opportunity to increase rates of HBV screening and immunization among women. Our data demonstrate the potential effectiveness of incorporating the triple screening tests into a prenatal order set (Appendix 2), as implemented at 2 of our obstetric clinic sites, to improve anti-HBs and anti-HBc testing among women, and such tests should be adopted by other obstetric sites to better adhere to CDC/ACOG guidance [1, 2]

## Supplementary Material

ofae632_Supplementary_Data

## Supplementary Data

Supplementary materials are available at *Open Forum Infectious Diseases* online. Consisting of data provided by the authors to benefit the reader, the posted materials are not copyedited and are the sole responsibility of the authors, so questions or comments should be addressed to the corresponding author.

## Notes

***Financial support*.** This work was supported by the Irma T. Hirschl/Monique Weill-Caulier Trust Research Award; and the National Institutes of Health (K23 HL163486-02 to T. K.).
