# Supplementary figures and images for: Hepatitis B Triple Panel Testing Implementation in the Obstetric Care Setting: Unique Predictors of Hepatitis B Virus Vaccine Immunity, Exposure, and Positivity
Source: Open Forum Infect Dis. 2024 Oct 23;11(11):ofae632. doi: 10.1093/ofid/ofae632 (PMC11558448; doi:10.1093/ofid/ofae632)

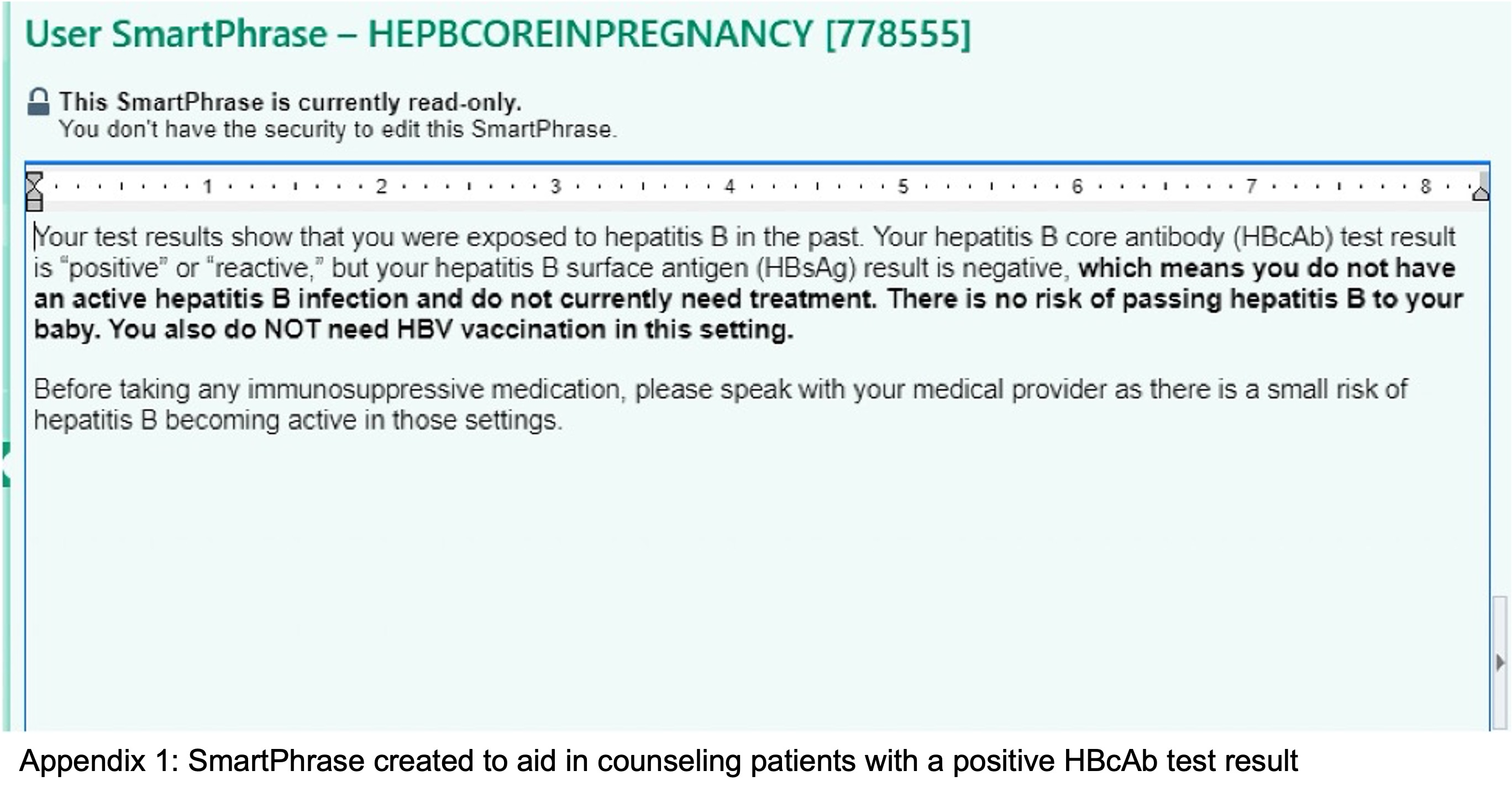

Supplement: ofae632_Supplementary_Data [file ofae632_supplementary_data.zip › AppendixFigure1_CID.tiff]

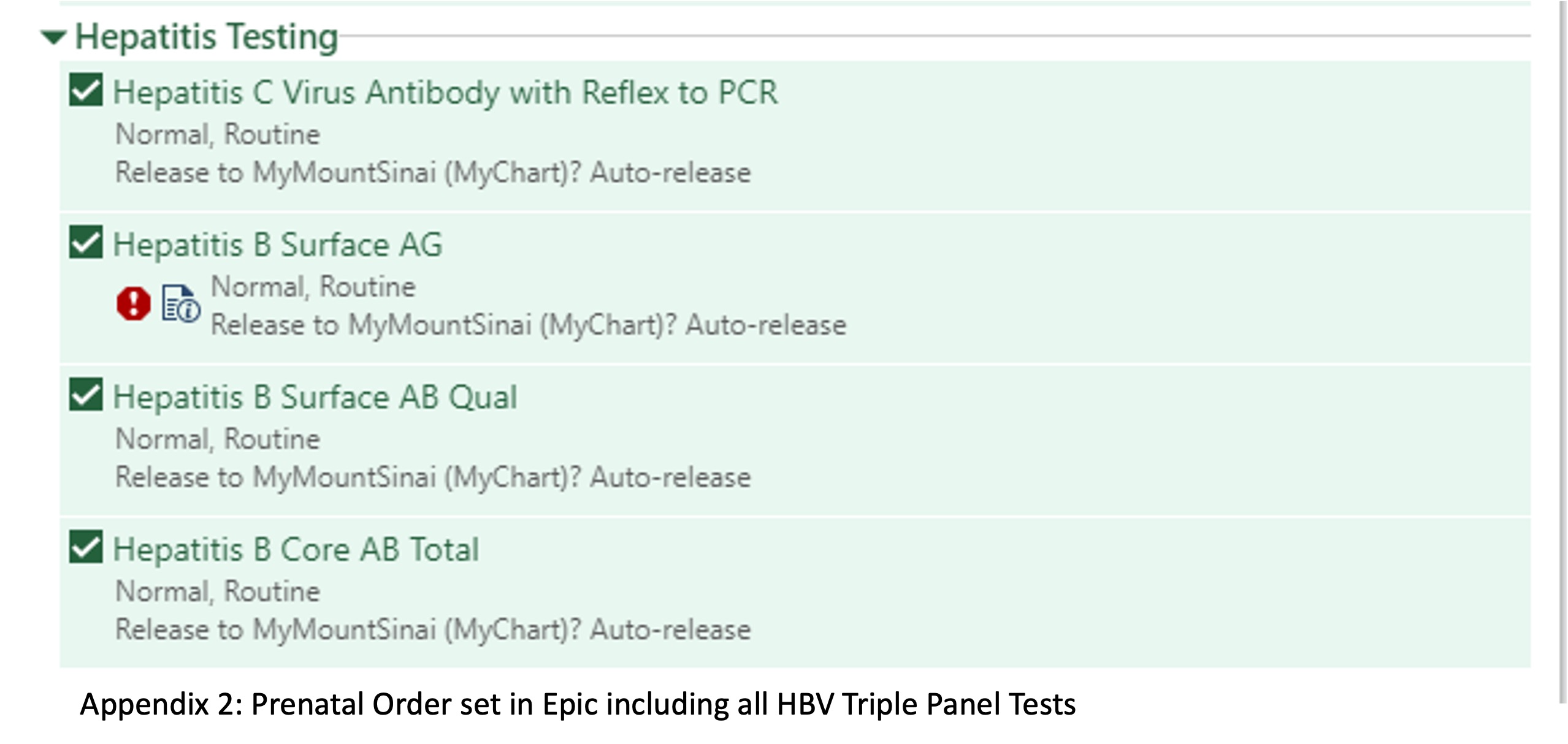

Supplement: ofae632_Supplementary_Data [file ofae632_supplementary_data.zip › AppendixFigure2_CID.tiff]
